# Mindfulness Training for Depressed Older Adults Using Smartphone Technology: Protocol for a Fully Remote Precision Clinical Trial

**DOI:** 10.2196/39233

**Published:** 2022-10-27

**Authors:** Abigail Schweiger, Thomas L Rodebaugh, Eric J Lenze, Katie Keenoy, Jason Hassenstab, Jeanne Kloeckner, Torie R Gettinger, Ginger E Nicol

**Affiliations:** 1 Healthy Mind Lab Department of Psychiatry Washington University School of Medicine Saint Louis, MO United States; 2 School of Social Work Saint Louis University Saint Louis, MO United States; 3 Department of Psychological and Brain Sciences Washington University in Saint Louis Saint Louis, MO United States; 4 mHealth Research Core Washington University School of Medicine Saint Louis, MO United States; 5 Trial Care Unit Center for Clinical Studies Washington University School of Medicine Saint Louis, MO United States; 6 Department of Neurology Washington University School of Medicine Saint Louis, MO United States; 7 Division of Child and Adolescent Psychiatry Washington University School of Medicine Saint Louis, MO United States

**Keywords:** mHealth, mindfulness, depression, aging, precision medicine, fully remote trial, technology, older, adult, smartphone, clinical trial, intervention, death, needs, preferences, online, remote, treatment, depressed, training, mind, session, medicine

## Abstract

**Background:**

Precision medicine, optimized interventions, and access to care are catchphrases for the future of behavioral treatments. Progress has been slow due to the dearth of clinical trials that optimize interventions’ benefits, individually tailor interventions to meet individual needs and preferences, and lead to rapid implementation after effectiveness is demonstrated. Two innovations have emerged to meet these challenges: fully remote trials and precision clinical trials.

**Objective:**

This paper provides a detailed description of Mindful MyWay, a study designed to test online mindfulness training in older adults with depression. Consistent with the concept of fully remote trials using a smartphone app, the study requires no in-person contact and can be conducted with participants anywhere in the United States. Based upon the precision medicine framework, the study assesses participants using high-frequency assessments of symptoms, cognitive performance, and patient preferences to both understand the individualized nature of treatment response and help individually tailor the intervention.

**Methods:**

Mindful MyWay is an open-label early-phase clinical trial for individuals 65 years and older with current depression. A smartphone app was developed to help coordinate the study, deliver the intervention, and evaluate the acceptability of the intervention, as well as predictors and outcomes of it. The curriculum for the fully remote intervention parallels the mindfulness-based stress reduction curriculum, a protocolized group-based mindfulness training that is typically provided in person. After consent and screening, participants download The Healthy Mind Lab mobile health smartphone app from the Apple App Store, allowing them to complete brief smartphone-based assessments of depressive symptoms and cognitive performance 4 times each day for 4 weeks prior to and after completing the intervention. The intervention consists of an introduction video and 10 weekly mindfulness training sessions, with the expectation to practice mindfulness at home daily. The app collects participant preference data throughout the 10-week intervention period; these high-frequency assessments identify participants’ individually dynamic preferences toward the goal of optimizing the intervention in future iterations.

**Results:**

Participant recruitment and data collection began in March 2019. Final end point assessments will be collected in May 2022. The paper describes lessons learned regarding the critical role of early-phase testing prior to moving to a randomized trial.

**Conclusions:**

The Mindful MyWay study is an exemplar of innovative clinical trial designs that use smartphone technology in behavioral and neuropsychiatric conditions. These include fully remote studies that can recruit throughout the United States, including hard-to-access areas, and collect high-frequency data, which is ideal for idiographic assessment and individualized intervention optimization. Our findings will be used to modify our methods and inform future randomized controlled trials within a precision medicine framework.

**Trial Registration:**

ClinicalTrials.gov NCT03922217; https://clinicaltrials.gov/ct2/show/NCT03922217

**International Registered Report Identifier (IRRID):**

DERR1-10.2196/39233

## Introduction

### Getting to Precision Medicine in Neurobehavioral Conditions: The Role of Smartphone-Based Technology

Precision medicine, that is, individually optimized treatment, needs to be the goal for clinical trials in neurobehavioral disorders [1]. The precision medicine target is particularly critical to depression, a highly heterogeneous condition in which individual treatments usually have small effect sizes at a group level.

To successfully develop precision medicine for depression, clinical trials of behavioral interventions need to overcome many causes of treatment failure at the individual patient level. In summary, these failures are the intervention not being a good fit for that particular patient’s symptom pattern (eg, proposing mindfulness training for depression when the driving force of the depressive symptoms would not respond to mindfulness), the intervention not being optimized to that individual patient (eg, does not provide content in the manner or frequency that the participant prefers or needs, leading to poor adherence or lack of benefit), and the assessment of the intervention outcomes (eg, depressive symptoms) having low reliability, leading to an apparent small treatment effect size. The precision clinical trial framework is a recently developed framework to overcome these obstacles to precision [1]. The framework explicitly develops and tests intervention optimization and personalization during a series of intervention development trials.

Another problem in clinical trials—especially those of behavior interventions—are their lack of reach and access (particularly to low-resourced individuals or those far from academic medical centers). This in turn leads to small and less generalizable study samples. Further, interventions tested only within academic medical centers tend to be implemented slowly if at all, a long-standing failure to translate science into practice [2]. Fully remote trials have been proposed as a way to overcome these problems by recruiting, screening, assessing, and treating patients where they live [3].

Smartphone-based technology can help drive the clinical trial innovations described above. In this study, we created an app designed to conduct a fully remote clinical trial of a behavior intervention—mindfulness for depression in older adults—and test it within a precision medicine framework. The study is the first phase in a line of research to both advance mindfulness as a precision medicine intervention for depression and advance clinical trial methodology.

### Mindfulness for Depression in Older Adults

Nationally, 18.4% of adults 65 years and older report symptoms of depression; 12% report subjective [4] cognitive decline [5,6]. Late-life depression and cognitive decline frequently coexist and can lead to Alzheimer disease and related dementias (ADRD) [7]. Given the growing older adult population and the rising public health concerns of late-life depression and ADRD, effective and accessible interventions are needed that ameliorate both depressive symptoms and cognitive functioning.

Mindfulness-based stress reduction (MBSR) may be an effective intervention for mitigating the effects of aging on the brain [8]. MBSR combines the teaching and practice of mindfulness meditation following an 8-week structured group-based protocol. Clinical trials indicate that MBSR practice may help in improving mood symptoms and neurocognitive performance in older adults [9,10]. Wetherell and colleagues [8] found that older adults who participated in an MBSR intervention group showed greater improvement in their mood and memory performance than those in a control group. Despite these promising findings, MBSR and other forms of mindfulness have still received limited testing in older adults, with few attempts at optimization as an intervention.

### Improving the Reach and Impact of Mindfulness

A traditional MBSR protocol requires frequent in-person contact, with participants attending 8 weekly mindfulness sessions that are 2 to 2.5 hours in length plus an all-day retreat. However, accessing in-person MBSR classes may be challenging for older adults given mobility concerns, physical comorbidities, transportation challenges, financial constraints, and geographic location. Mobile health (mHealth) technology provides a mechanism for reaching individuals who are unable to access or attend in-person study visits and mindfulness training [11]. Even prior to the onset of the COVID-19 pandemic, many people expressed a preference for individual and online mindfulness training over in-person group formats [12]. Videoconferencing-based treatments have shown promise for older, rural, and minority adults [13].

### Smartphone-Based Assessments for Precision Intervention Development

Although the terminology itself is somewhat imprecise, the contemporary understanding of the term *precision medicine* refers to increasingly granular aggregate data that provides mechanistic insight into either the manifestation of illness, response to a treatment intervention, or both [14]. Originally rooted in pharmacogenomics, precision medicine methods and practices were first integrated into clinical care in medical subspecialties like oncology, where biomarkers are proximally linked to illness or treatment response [15]. In the last decade, digital phenotyping, which incorporates data collected in real time from mobile devices like smartphones and wearable sensors, offers precision measures of individual behaviors, characteristics, and related contextual factors [16]. These data elements can then be examined to identify which ones significantly drive treatment engagement over time, and how they can be optimized to enhance intervention effectiveness [17]. Building upon precision medicine methods and practices, delivery of behavioral interventions such as mindfulness training for older adults would ideally use both biomarkers and digital phenotyping. Identifying needs and preferences that drive engagement would allow for more precise intervention delivery, thereby leading to improved outcomes.

However, our ability to reliably identify and detect valid markers that predict symptoms, treatment engagement, and response remains limited for neuropsychiatric disorders due, in part, to inexact outcome assessment strategies in clinical trials [1,18,19]. For example, standard outcome measures of depression symptom severity and treatment response provide a unidimensional and static view of the individual’s unique characteristics, needs, and preferences that in reality are dynamic and highly dependent on contextual factors over time. However, recent advances in mobile and wearable technology make conducting *N*=1 analyses possible by generating multiple data points that enhance the detection of group- and individual-level patterns in symptoms, needs, and preferences over time, allowing for the prediction of symptom dynamics (eg, tendency to increase, sustain, or cycle), as well as user engagement (eg, how and for how long users interact with the intervention) [20,21].

### Study Aims

Engagement of the target population in the app creation process is critical for developing scalable and effective mHealth interventions for this population [22]. A key factor influencing older adults’ use of mHealth technology is the intervention’s usability. Usability considers the extent to which the technology (eg, app) can be used by the target-specified population (eg, older adults) to achieve goal effectiveness, efficiency, and user-satisfaction benchmarks [23]. Yet older adults are rarely consulted in the design of technologies for health [24]. This may affect the adoption of the technology, adherence, optimization of treatments, and desired outcomes, thereby limiting the ability to achieve precision medicine or individually optimized treatment. Thus, it is necessary to engage the target population in testing the usability of mHealth interventions.

Here, we describe the development and early usability testing of the Mindful MyWay program and The Healthy Mind Lab mHealth app for older adults with depressive symptoms. This online internet-delivered mindfulness meditation course is comprised of prerecorded videos of mindfulness education and practice recommendations (eg, meditation) that a participant can watch on their computer or mobile device at their convenience. The study app not only serves to link participants to upcoming mindfulness classes and prompt daily home meditative practice but also deploys frequent scheduled daily assessments of depressive and cognitive symptoms. The frequent outcome assessment data will inform the development of future *N*=1 analytic models, which are critically needed for the successful development of self-adaptive algorithms for personalized digital interventions. As well, the app conducts daily needs assessments. These assessments evaluate barriers and ask questions about ways to enhance the participants’ mindfulness practice. This provides user needs and preference data for future precision development of the mindfulness intervention.

### Study Objectives

This study has the following objections:

- Objective 1: To assess the usability, feasibility, and acceptability of internet-delivered mindfulness sessions and overall usability of the smartphone app
- Objective 2: To collect high-frequency ecological momentary assessment (EMA) data, such as depression symptoms and cognitive testing data from participants pre- and postintervention, and high-frequency patient preference data during the intervention
- Objective 3: To assess temporal dynamics (within-individual changes and interrelationships over time) of depressive symptoms

## Methods

### Study Procedures

**Figure 1:**
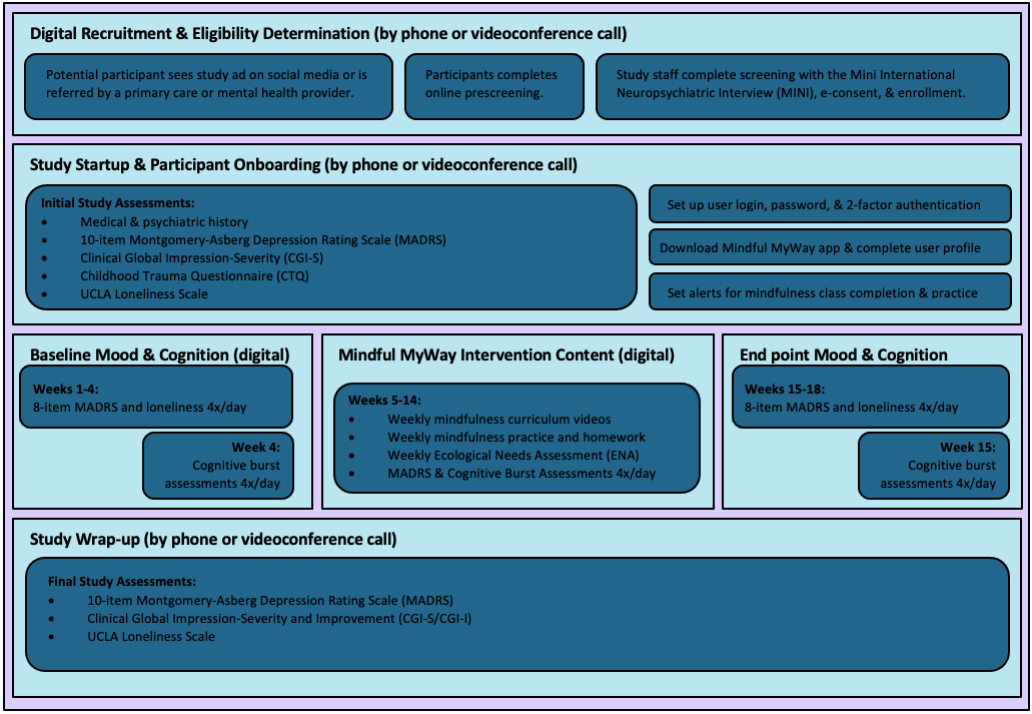
Flowchart indicating the study procedures and mindfulness training intervention phases.

A flowchart with the study procedures and mindfulness training intervention phases is shown in Figure 1.

### Ethics Approval

The Mindful MyWay study was determined to present no more than minimal risk of harm to participants and involves no procedures for which written consent is normally required outside of the research context (45 CFR 46.117(c)(ii)/ 21 CFR 56.109(c)(1)(ii)). The study was approved and received a waiver of documentation of consent from the Washington University Institutional Review Board (IRB) on February 26, 2019 (IRB ID# 201811036).

### Recruitment

Older adults aged ≥65 years with a mobile device using iOS 6 or higher who are experiencing a current depressive episode are invited to participate. Participants are excluded if they are unable to provide informed consent, have a known neurological condition that may confound the interpretation of results (eg, stroke, seizure disorder, or multiple sclerosis), are actively engaged in cognitive therapy or training, or are expressing acute suicidal or homicidal ideation. Participants are recruited from the Washington University Center for Mental Health and Wellness geriatric psychiatry clinic, a network of community outpatient primary care and psychiatry clinics used in previous clinical trials, and the institutional volunteer registry.

### Screening

After completing a digital questionnaire accessed via a REDCap survey link sent in a secure email, a research team member contacts the potential participant by phone to conduct a screening interview and determine eligibility. For participants who qualify after screening, the research team member provides the instructions for downloading the Mindful MyWay smartphone app onto a mobile device using iOS version 6 or newer. Instructions for downloading and using the app are also emailed to the participant for future reference.

### Electronic Informed Consent

This study was approved by the Washington University IRB as a minimal risk study and granted a waiver of written consent. Participants meeting eligibility criteria and passing prescreening procedures receive a digital consent document via email to review. Study staff schedule a time to review the consent document with the participant and answer any questions. Once all study-related procedures, risks, and benefits are explained and discussed, participants provide verbal consent to participate in the study.

### Smartphone-Based Intervention and Assessments

**Figure 2:**
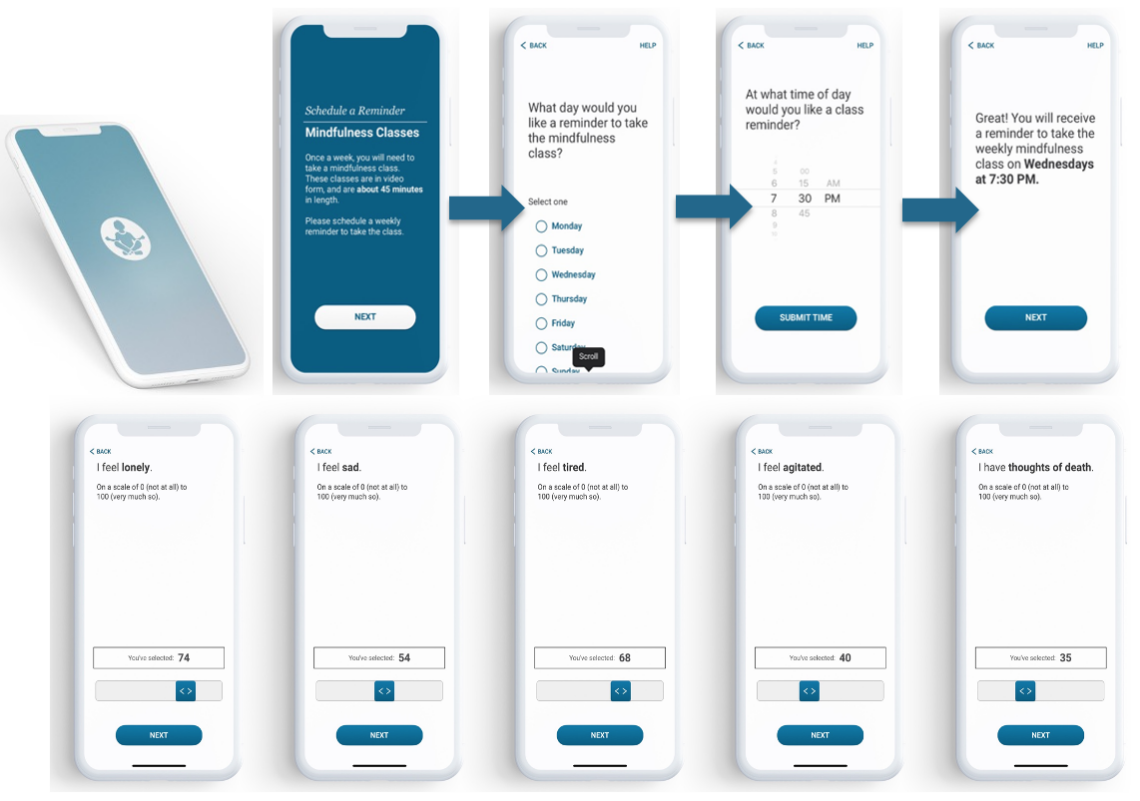
Mindful MyWay app introductory screens. (A) The top row is an example of the introductory screens that allow participants to choose an individualized day and time for their class reminders. (B) The bottom row is an example of ecological momentary assessments sent to the participants 4 times each day.

Figure 2 shows features of the smartphone app developed for the study. This app was designed based on prior studies completed by our group using smartphone-based assessments to evaluate the effects of mindfulness interventions for older adults [25-28]. This study was inspired by qualitative work completed with participants in our previous studies indicating that digital self-guided access to mindfulness training and practice would increase the likelihood of engaging in mindfulness activities with the aim of improving mood and cognitive functioning [29]. The resulting app represents the minimum viable digital framework for promoting mindfulness practice in older adults by delivering digital mindfulness training, which includes 10 weekly videos that are based on the MBSR curriculum developed by Kabat-Zinn [30], providing cues to engage in and track mindfulness practices. The online MBSR course videos are housed on YouTube, accessible only to participants via the study Qualtrics website, and includes 10 weekly prerecorded videos, approximately 45 minutes each.

Prior to beginning the Mindful MyWay study, a member of the research team guides the participant through the app download and 2-factor authentication log-in process. Participants are then asked to indicate windows of time during waking hours when they prefer to complete study assessments (Figure 2A). Participants are also mailed a workbook containing information about each session and corresponding activities, home practice suggestions that can be completed between weekly video sessions, and a glossary of terms.

During the initial app setup, participants are asked to schedule a time each week to complete the video lesson. They receive a smartphone push notification containing a URL hyperlink to an external website with a new MBSR video each week during the study. Study staff can monitor participant engagement via the Qualtrics dashboard, which tracks the completion of scheduled digital assessments and alerts study staff when assessments are missed. Study staff checks in weekly with participants via text, email, or phone to encourage continued participation and answer questions or, more frequently, to follow up on any missed assessments or critical depression scores.

Depression is assessed using a Modified Montgomery-Asberg Depression Rating Scale (MADRS) assessment, which includes eight items derived from the MADRS [25] (sadness, agitation, fatigue, concentration, motivation, anhedonia, pessimism, and thoughts of death). In June 2019, we added an EMA item measuring loneliness. The rationale for this addition was that a mindfulness intervention could reduce loneliness in older adults [31], and this study could provide critical feasibility data, such as whether EMA measurement of loneliness was feasible and whether the mindfulness intervention as designed would target loneliness or that the data (either lack of improvement or participant self-reported preferences) would indicate the need for enhancement of the intervention. The loneliness EMA item added was “I feel lonely (0, not at all, 100, very much so).” Mood assessments are conducted four times daily within 4-hour intervals during waking hours designated by the participant at study enrollment. Responses are on a visual analog scale with scores ranging from 0 (not at all) to 100 (very much so; Figure 2B).

Self-administered neuropsychological testing uses the smartphone-based Ambulatory Research in Cognition rapid burst cognitive assessments created by Dr Jason Hassenstab for the Dominantly Inherited Alzheimer’s Network (DIAN) study with the Washington University DIAN Trials Unit [32]. The goal of this high-frequency neuropsychological assessment, sometimes called “burst cognitive sampling,” is to increase the validity and reliability of the measurement of cognitive impairment [33]. Participants provide preferred windows of time during waking hours in which to receive 4 prompts each day to complete assessments for the 7 days prior to starting the intervention and for 7 days after completing the intervention. Prior to each assessment, data about the participant’s immediate environment is collected (eg, where they are, what they are doing, and who they are with). The assessment battery is comprised of three separate tests conducted in a randomized order. These include tests for spatial working memory, processing speed, and associate memory with the Grids, Symbols, and Prices tasks, respectively. Each assessment presents the individual tests in a randomized order and takes approximately 2 minutes to complete.

To identify drivers of engagement with the app, participant preferences are collected via qualitative EMAs referred to as ecological daily needs assessments (EDNAs) [26]. EDNAs are brief questions that evaluate intervention feasibility and acceptability in real time, revealing factors specific to the digital context that serve as facilitators or barriers to engagement. For the purposes of the Mindful MyWay app, engagement was defined as viewing weekly mindfulness training videos (via clicks on the website that houses the mindfulness video content) and practicing new mindfulness skills (via self-report) during the 10-week intervention period. Participants are asked each day, at random times, whether they completed the mindfulness practice and homework as prescribed (eg, “How much of the day today did you feel you were ‘present’ or ‘mindful?’”), what type of mindfulness practice was used (eg, “If you spent time being mindful today, select which practices you used and how much time you spent.”), and feedback about what would make the app more helpful (eg, “What would help you be mindful right now?”). EDNA data is collected during pilot-testing with the intent of discovering whether modifications to the timing of assessment prompts, feasibility and acceptability of active tasks, and appropriateness of intervention content are needed to optimize engagement. These data also inform the development of digital algorithms for future adaptive iterations of the intervention.

### Other Assessments

These assessments are administered by study staff with participants via survey or phone at baseline and after completing the 10-week study intervention.

The Mini-International Neuropsychiatric Interview (MINI) [34] is a short-structured diagnostic interview developed for *Diagnostic and Statistical Manual of Mental Disorders* (Fifth Edition; *DSM-5*) [35] psychiatric disorders and takes approximately 45 minutes to complete. In this study, the MINI is conducted by a trained member of the research team via a videoconference call during screening to confirm eligible diagnoses and to rule out any exclusionary *DSM-5* diagnoses.

The MADRS is a 10-item measure administered by a trained research staff member to assess depressive symptom severity. In this study, the 10-item MADRS is administered over the phone by a trained member of the research team via videoconference call at baseline and the end point (week 18). The UCLA Loneliness Scale is a 20-item self-report measure that assesses how often a person feels disconnected from others [36]. Participants complete this assessment online at baseline and the end point. This assessment was added at the beginning of the COVID-19 pandemic to collect data on the impact of isolation and loneliness on this older depressed population.

### Plans for Data Analysis

We will analyze feasibility with respect to retention and completion rates, successful and unsuccessful recruitment methods, and successful and unsuccessful methods to prompt adherence to the intervention, including home practice of mindfulness. We will also examine participant feedback gathered via the daily needs assessments throughout the intervention period; we will examine not only group-level data but also individual data over time to ascertain whether participant needs are individually dynamic (eg, one participant has one set of needs that changes over time during the intervention period, whereas another participant has different needs that do not change in a different pattern).

With respect to predictor and outcome data, we will examine these in several ways to generate hypotheses for testing in future trials. In short, multilevel dynamic structural equation modeling (ML-DSEM) will be used because of its suitability for intensive longitudinal data [37]. In the primary model, the treatment effect will be captured as a pre-post contrast in overall response to EMA depression items, allowing the identification of individuals who show significant response versus those who do not. Those who show significant response can then be characterized via the other measures in the study. Other applications of ML-DSEM to treatment data (eg, as suggested by Hamaker and colleagues [38]) and the *N*=1 application of the model will be examined to yield potential insights into predictors of treatment response. The cognitive data will be averaged at baseline and postintervention to calculate a reliable assessment of cognitive status at each time point; in addition, we will assess the test-retest reliability of these cognitive data.

## Results

The study was approved and received a waiver of documentation of consent from the Washington University IRB on February 26, 2019. After prescreening 151 potential participants, 31 verbally consented between March 28, 2019, and April 7, 2022, with 23 participants included in the intention-to-treat group. The mean age of participants was 71.7 (SD 4.5) years; 16 (85%) were women, and 1 (5%) was non-White. Results are expected in July 2022.

## Discussion

### Limitations and Directions for Future Research

One of the main challenges we faced in this study was creating a user-friendly app that reinforced participants’ positive attitudes toward mHealth app use. Patient stakeholders are involved in the assessment of this app as part of the feasibility testing but were not involved in the initial development of the app. Future iterations of the study intervention and design of the app should incorporate a user-centered design with stakeholder input, particularly from patients. This is especially important as older adults with depression may have unique technology use challenges and benefit from reinforcements to enhance adherence. Therefore, interventions should be developed in collaboration with the end user and consider factors related to usability and appropriateness of the intervention, as well as user preference, needs, and engagement. Likewise, “the scientific community will benefit from continued funding opportunities and academic research institutions adopting a digital infrastructure to support investigators in using and navigating mobile technology for clinical research and healthcare delivery” [39].

### Conclusions

The Mindful MyWay study exemplifies the innovative clinical trial designs that can be conducted using smartphone technology. By using an app and internet-based video content, supplemented by phone and other distance-based contact from research staff, we are conducting a fully remote clinical trial. The entire study, from screening to outcome completion, is done at a distance; this increases reach and access of clinical trials, as well as the evidence-based interventions that ensue from them.

In this study, high-frequency data collection of outcome and participant feedback, together with advanced analytic approaches, will lead to more precise and individually relevant outcome assessment, as well as personalized intervention optimization. Our findings will be used to modify our methods and inform future randomized controlled trials within a precision medicine framework.

This study is being conducted in depressed older adults with a mindfulness intervention. However, the broad methods being used are widely applicable to behavioral intervention research, especially for neurobehavioral conditions such as depression in which a precision medicine approach is needed to advance the science of interventions. As the COVID-19 era has shown us that fully remote interventions and trials are indeed feasible, we expect more such research in the future.

## Abbreviations

ADRD: Alzheimer disease and related dementias
DIAN: Dominantly Inherited Alzheimer’s Network
DSM-5: Diagnostic and Statistical Manual of Mental Disorders (Fifth Edition)
EDNA: ecological daily needs assessment
EMA: ecological momentary assessment
IRB: Institutional Review Board
MADRS: Modified Montgomery-Asberg Depression Rating Scale
MBSR: mindfulness-based stress reduction
mHealth: mobile health
MINI: Mini-International Neuropsychiatric Interview
ML-DSEM: multilevel dynamic structural equation modeling

## Data Availability

All data requests should be directed to the corresponding author.
